# Detailed evaluation of cancer sequencing pipelines in different microenvironments and heterogeneity levels

**DOI:** 10.3906/biy-2008-8

**Published:** 2021-04-20

**Authors:** Batuhan Kısakol, Şahin Sarıhan, Mehmet Arif Ergün, Mehmet Baysan

**Affiliations:** 1 Department of Physiology and Medical Physics, Centre for Systems Medicine, Royal College of Surgeons in Ireland, Dublin Ireland; 2 Computer Engineering Department, Faculty of Engineering, Marmara University, İstanbul, Turkey Turkey; 3 Computer Engineering Department, Faculty of Computer and Informatics Engineering, İstanbul Technical University,İstanbul Turkey

**Keywords:** Clinical bioinformatics, next generation sequencing, cancer, mapping algorithms, variant discovery algorithms

## Abstract

The importance of next generation sequencing (NGS) rises in cancer research as accessing this key technology becomes easier for researchers. The sequence data created by NGS technologies must be processed by various bioinformatics algorithms within a pipeline in order to convert raw data to meaningful information. Mapping and variant calling are the two main steps of these analysis pipelines, and many algorithms are available for these steps. Therefore, detailed benchmarking of these algorithms in different scenarios is crucial for the efficient utilization of sequencing technologies. In this study, we compared the performance of twelve pipelines (three mapping and four variant discovery algorithms) with recommended settings to capture single nucleotide variants. We observed significant discrepancy in variant calls among tested pipelines for different heterogeneity levels in real and simulated samples with overall high specificity and low sensitivity. Additional to the individual evaluation of pipelines, we also constructed and tested the performance of pipeline combinations. In these analyses, we observed that certain pipelines complement each other much better than others and display superior performance than individual pipelines. This suggests that adhering to a single pipeline is not optimal for cancer sequencing analysis and sample heterogeneity should be considered in algorithm optimization.

## 1. Introduction

Cancer is a major threat to human health that leads to millions of deaths each year worldwide (Siegel et al., 2019). This malady is well-documented in human history since ancient Egyptian civilization and remains a major adversary of humanity (Mukherjee, 2010). Cancer is a result of the transformation of cells through which they obtain uncontrolled growth. Understanding the molecular changes that lead to this transformation is critical to prevent and treat cancer. Changes in chromosomal abnormalities were observed in cancer since the early studies of Boveri at the beginning of the 20th century (Baltzer, 1964). Recent improvements in sequencing technologies made the fast and inexpensive mapping of cancer genomes possible (Tucker et al., 2009). This was a major step in cancer research and led to large-scale cancer genome profiling studies such as The Cancer Genome Atlas (TCGA) (Weinstein et al., 2013) and International Cancer Genome Consortium (ICGC) (Zhang et al., 2011). These studies confirmed the existence of mutations that arise in parallel to tumor formation and identified recurrent mutations that potentially drive increased cell proliferation and motility.

Massively parallel (next-generation) sequencing technologies are based on shredding DNA into small fragments and determining the nucleotide sequence in these fragments. These short reads are mapped to the reference genome to identify the genome sequence of the sample. In cancer samples, genome sequences from tumor and nontumor tissue belonging to the same individual are compared to find cancer-specific somatic mutations. These two steps (mapping and variant calling) constitute the two major steps of cancer sequencing analyses, and many algorithms were developed for these tasks. These algorithms are combined in software pipelines, which take raw sequencing data as input and produce cancer-specific changes as output. Different mapping and variant discovery algorithms have different assumptions and priorities. As a result, the number and type of variants identified by these algorithms might vary significantly. This makes detailed testing of constructed pipelines for different situations an essential task for efficient utilization of sequencing technologies.

Benchmarking sequencing pipelines is an active field of research. Extensive studies on the performances of aligners and variant callers have been carried out (Hatem et al., 2013; O’Rawe et al., 2013; Pirooznia et al., 2014; Hwang et al., 2015; Hwang et al., 2019). Hwang et al. (2019) comprehensively evaluated a combination of 7 mapping and 10 variant calling algorithms using high confidence variants as validation datasets from three different platforms. The authors suggested that the choice of variant calling algorithm is crucial. Hwang et al. (2015) analyzed discordant variant calling results on different datasets. They proposed to use different pipelines for different datasets. Due to the availability of precise evaluation metrics, these studies concentrate on germline mutation callings.

In addition to these works, several studies evaluate somatic mutation calling with tumor-normal samples (Wang et al., 2013; Hofmann et al., 2017; Ellrott et al., 2018; Chen et al., 2020). However, unlike germline calling tests, somatic variant callers lack a strict ground truth data set. Thus, different studies have followed different methodologies to overcome this problem. Some studies used simulated samples to measure the efficiency, some others used Sanger sequencing for validation (Roberts et al., 2013; Cai et al., 2016; Krøigård et al., 2016; Bohnert et al., 2017). A review on variant callers by Xu (2018) showed the potential biases and dependencies of these methods and datasets. The study suggested that a collection of real cancer genomes with high confidence variant datasets could greatly benefit benchmarking studies. Moreover, most of the benchmarking studies in cancer sequencing focus on variant callers but not aligners.

In an open-source cloud project, Ellrott et al. (2018) compared 10 variant callers on 10,510 tumor/normal pairs from 33 cancer types in the TCGA collection of whole-exome sequencing data. In total, the data set contained around 4 million variants after filtering nonexonic and possible germline variants. The authors used a different pipeline procedure to select validated variants like applying allele fractions and read count thresholds from different data types (WGS, WXS, Targeted, and RNA). Alioto et al. (2015) conducted a benchmarking study on lymphocytic leukemia and medulloblastoma tumor/normal samples for whole-genome sequencing data. They recommend optimizing aligner/variant caller combination and combining multiple variant callers. Hofmann et al. (2017) examined different variant callers and aligners for whole-genome sequencing datasets. Their validation method was generated by nine variant calling algorithms in simulated kidney tumor datasets. This study tested VC algorithms in different coverage levels and compared variant allele frequencies. Besides, the authors reveal that a combination of different pipelines outperforms a single pipeline.

Here, our study takes into account the effect of aligners and variant callers in cancer sequencing samples at different heterogeneity levels. We also analyzed the effect of pipelines combinations, where we combined three mapping (Bwa (Li and Durbin, 2009); Bowtie2 (Langmead and Salzberg, 2012); and Novoalignhttp://www.novacraft.com/products/novoalign/[accessed 23 08 2020]) and four variant calling algorithms (Mutect2 (v4.1.0) (Cibulskis et al., 2013); Varscan (v2.3.9) (Koboldt et al., 2012); SomaticSniper (Larson et al., 2011); Strelka2 (v2.9.10) (Saunders et al., 2012)). This resulted in twelve mapping-variant calling combinations labeled as “Bwa_Mutect2, Bwa_Varscan” etc. Although these algorithms were introduced a long time ago and more recent algorithms exist, these algorithms get updated regularly and are still considered as the most frequently used, state of art models. A recent paper, that reviews the best practices for variant calling in clinical sequencing, lists most practiced and cited variant callers which includes all the variant callers in this article (Koboldt, 2020). Several other latest articles, which compares the performances of variant calling pipelines in various scenarios, have chosen to benchmark VarsScan2, Mutect2 and Strelka2 (Wang et al., 2019; Chen Z et al., 2020). Therefore, we chose to evaluate the most commonly practiced workflows.

Recently, we have published a dataset of fifty-five high-resolution homogeneous and heterogeneous glioblastoma samples. (Baysan et al., 2017). These samples share a substantial portion of mutations which allows us to declare these mutations as validated mutations; since for an algorithm identifying a non-existing mutation twice in two independent samples is very unlikely. This dataset had four different sample types: (i) primary tumor samples from different parts of a glioblastoma tissue block, (ii) in vitro polyclone samples cultured from tumor stem cell lines of the primary tumor, (iii) in vivo polyclone samples obtained from mouse xenografts after in vitro polyclones were injected to a mouse brain and formed a tumor, (iv) in vitro monoclone samples obtained from in vitro polyclone samples through isolation of single cells and subsequent culturing until there are enough cells for exome sequencing. The availability of these samples presented us with a unique opportunity to test sequencing pipelines at different heterogeneity levels. For the mentioned pipelines, first, we compared the mutation lists through pairwise comparisons. Mutations were declared “validated” when they were detected in two independent samples. Validated mutations were used to evaluate the performance of each pipeline and different pipeline combinations.

Multiple studies have also been conducted on somatic indel variant callers, as well (Hasan et al., 2015; Kim et al., 2017; Chen et al., 2020). However, due to a couple of reasons, accurate indel classification is far more challenging than SNVs. First of all, aligners tend to map reads to multiple mismatches rather than indel sequences because of the short read lengths (Ghoneim et al., 2014). Secondly, false positive rates are higher compared to SNVs as a result of both read distribution and identical repetitive sequences (Narzisi et al., 2014). A study done by Fang et al. (2014), shows that indel and structural variant detection in exome-sequencing is relatively unreliable. In another article, inter-caller agreement of 5 variant callers on indel variants is as low as 0.01% which proves the inconsistency (Krøigård et al., 2016). Due to these reasons, only SNVs were included in this study. 

We have also simulated realistic samples with known mutations and compared these results with our original samples. In order to observe the effect of mutation frequencies on variant detection, we have generated tumors with low, medium, and high tumor purity.

## 2. Materials and methods

### 2.1. Samples

Sequenced samples were generated with Illumina paired-end sequencing technology at Ambry Genetics. Each paired-end read comprised of 8 million reads, 100bp read length and mean quality scores were between 34 and 38. Supplementary Figure 1 shows an example of standard read quality and read length. The dataset was consisted of a total of 50 glioblastoma samples; primary tumors from seven regions of a single tumor, 13 in vivo polyclones, seven in-vitro polyclones, 19 in vitro monoclones, and four secondary monoclones. A matched blood sample was sequenced as a matched normal sample. All sequenced reads were mapped to the human reference genome version GRCh38. The coverage of samples was between 50×–100× (Baysan et al., 2017). Randomly selected four samples’ coverage distribution with different mapping tools were shown in Supplementary Figure 2.

**Figure 1:**
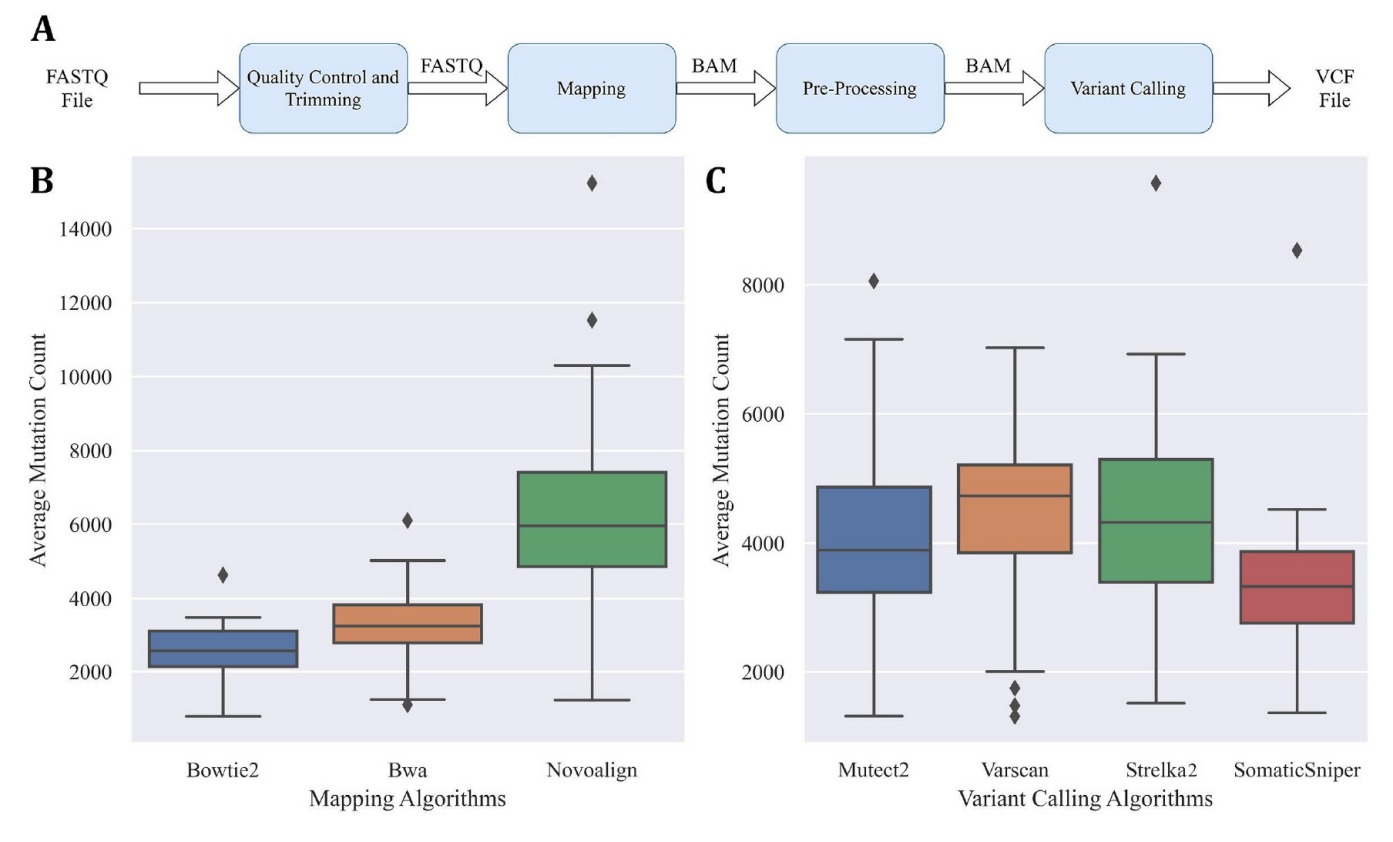
(A) The workflow of a simple Cancer DNA-Seq analysis pipeline including the steps and input/output files. (B) Boxplot of average mutation counts by the mapping algorithms. Y-axis represents the average variant number. Since 4 variant callers were used, the mean of these four variant callers with the same mapping algorithm represents the average value for each sample. The X-axis shows the corresponding mapping algorithm. (C) Boxplot of average mutation counts by the variant callers. Y-axis represents the average variant number. Since 3 mapping algorithms are used, the mean of these three mapping algorithms with the same variant caller represents the average value for each sample. The X-axis shows the corresponding variant callers.

**Figure 2:**
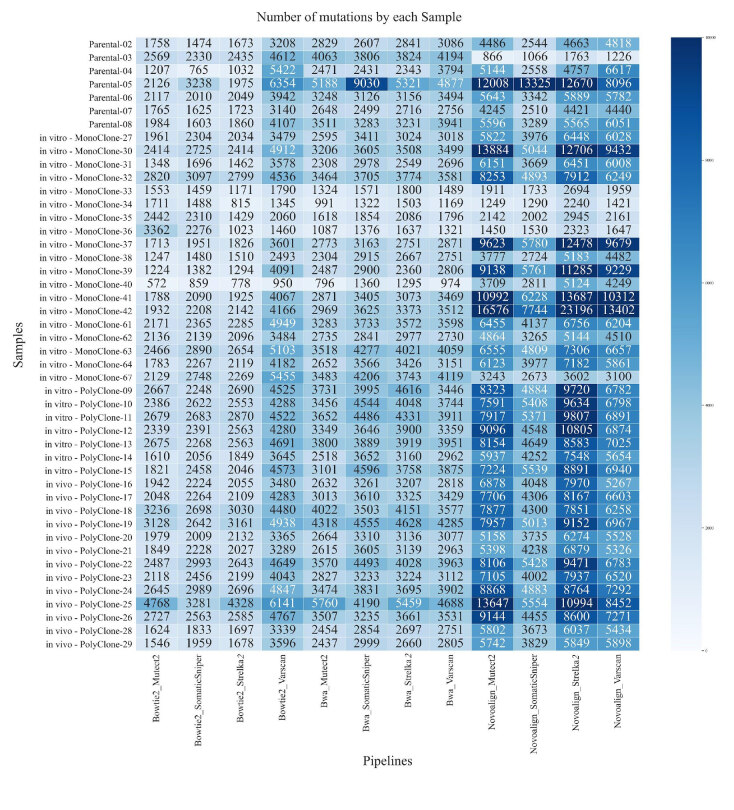
Heat map of the number of detected variants for each sample by the pipelines. Rows represent the samples and columns represent pipelines. The color scale displays the total number of variants found by the pipeline in a sample (dark color indicates more variants found and light color indicates fewer variants found).

### 2.2. Pipelines

A typical cancer sequencing pipeline includes following steps; quality control and trimming, mapping, preprocessing and variant calling. In this study, we created a set of pipelines for which FASTQ formatted raw sequenced data was used as input. The output of each pipeline was a variant containing VCF file (Figure 1(A)). The pipeline software was implemented in Python language and can be downloaded from our GitHub pagehttps://github.com/MBaysanLab/GenomicsPipeline [accessed 23 08 2020]. Each pipeline uses different algorithms for mapping and variant calling steps. All other steps and tools in the pipelines were identical, namely quality control of reads via FastQChttps://www.bioinformatics.babraham.ac.uk/projects/fastqc [accessed 23 08 2020], trimming by FASTP (Chen et al., 2018) and SAMTools (Li et al., 2009), and preprocessing according to recommended best practices using GATK4 (Van der Auwera et al., 2013).

Pipelines include three alternative mapping algorithms namely BWA, Bowtie2, and Novoalign and four alternative variant calling algorithms i.e. Mutect2, Varscan, SomaticSniper, and Strelka2. Each pipeline was designed to contain a combination of one mapping and one variant calling algorithm. Therefore, twelve pipelines were created to represent all combinations (Supplementary Table 1). All algorithms were used with their default parameters to evaluate their performance with their default setup. For the sake of fairness, minimum variant read depth was fixed to 10 for each pipeline.

### 2.3. Simulating tumor samples

To compare our results in the real dataset to an experimental simulation, we generated simulated reads using NEAT read simulator (Stephens et al., 2016). This tool generates FASTQ and VCF files with tumor/normal samples, thus enabling us to extend the scope of our benchmarking study. We utilized the same exome regions in the real dataset and pooled all the variants detected by any of our pipelines in the real samples. NEAT generates reads with random mutations by using this variant pool and exome regions. We created around 4800 variants in three different purity environment. 

First, we constructed a normal (nontumor) FASTQ file with 80× coverage, then created two different tumor files with 20× and 80× coverages. The 20× and 80× tumor files contains 4864 and 4764 mutations, respectively. By combining 20× tumor file with 80× normal reads, firstly tumor file is created with %20 tumor purity. Then 80× tumor sample was merged with 80× normal sample and produced a tumor with 50% purity. Lastly, only the 80× tumor sample was used without merging with the normal sample to have a sample with 100% tumor purity. By extracting the variants presented in the tumor VCFs, we constructed the list of the synthetic somatic variants. All three purity environments share the similar number of mutations that are produces by the same mutation model inferred from the original variant pool. Thus, in this simulation we investigated how different pipelines perform finding mutations in different tumor purities.

## 3. Results

Each of the twelve pipelines was applied on 50 tumor samples, which resulted in 600 runs in total. We evaluated the pipelines according to the number of variants found. Pipelines that used Novoalign as the mapping algorithm found more variants than BWA and Bowtie2 pipelines. Among all, Bowtie2 pipelines had the least number of discovered variants (Figure 1(B)). When we group samples by variant calling algorithms, SomaticSniper found the least number of variants while Strelka2, and Varscan found more variants (Figure 1(C)). Primary samples (except Parental-5) have fewer mutations compared to other samples, which is expected due to the high level of heterogeneity and potential nontumor contamination in these samples. We also observed a similar pattern in our four monoclone samples (Figure 2). Since the overall number of variants was smaller in these samples, the number of validated variants were also less.

**Figure 3:**
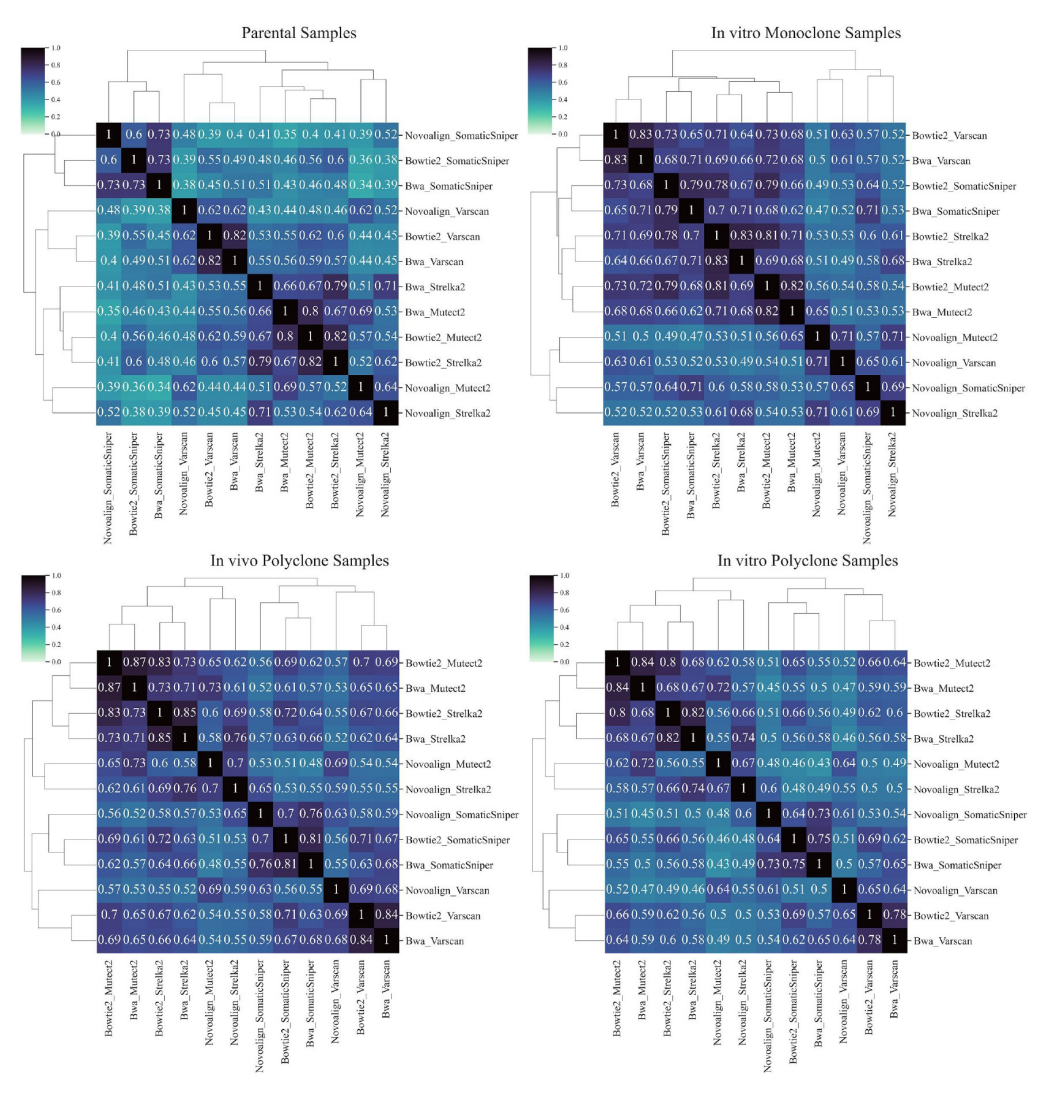
Clustered similarity matrices for each sample type. Cosine similarity values are represented in the cells along with the color scale (dark colors ‒ higher cosine similarity and light colors ‒ lower cosine similarity among pairs). Dendrograms indicate hierarchical clusters among pipelines. Thus, pipelines with a higher similarity clustered together.

Next, we analyzed the similarities of identified variants between different pipelines on different sample types. In Figure 3, cosine similarity matrices for each pipeline for each sample type are displayed (see Supplementary Figure 3 and Supplementary Figure 4 for Pearson and Jaccard correlations). In primary tumor and polyclone samples (both in vitro and in vivo), pipelines that included SomaticSniper and Varscan as variant discovery algorithms clustered together regardless of the applied mapping algorithm. Pipelines that included Strelka2 and Mutect2 produced most of the time similar results when used with the same mapping algorithm. On the other hand, in monoclone samples, pipelines that used Novoalign clustered together and were clearly separated from the rest. For the pipelines that used BWA and Bowtie2 as alignment algorithms, variant discovery algorithms were the dominant factor with respect to clustering.

**Figure 4:**
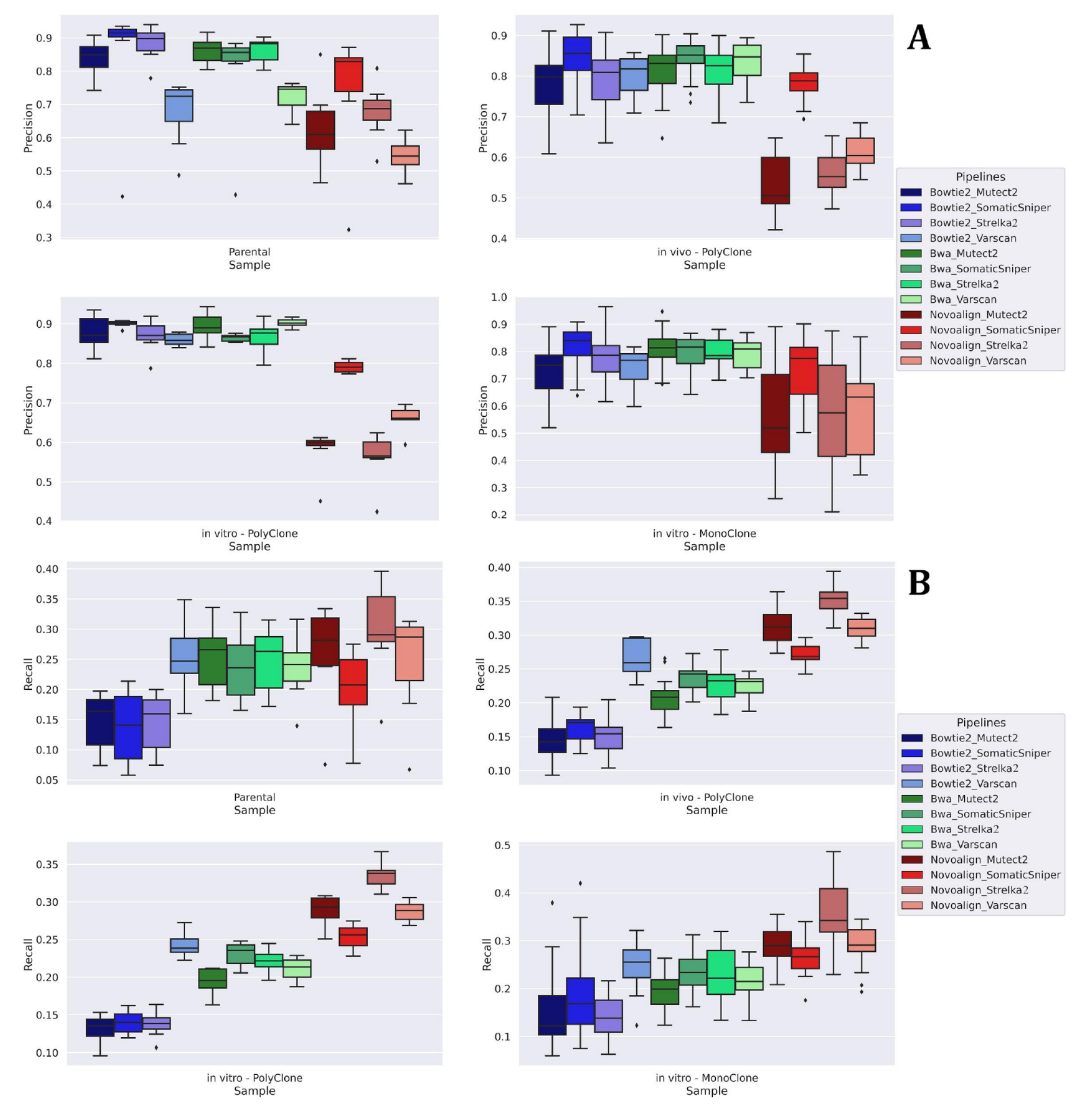
Precision (A) and recall (B) box plots for individual samples in each sample type.

After the comparison of the obtained variants for different pipelines, we utilized the multiple detections of variants in different samples as a validation method. Based on these validated variants, precision, recall, and F1 scores were computed for each pipeline (Figure 4). A true positive (TP) variant is defined as the detection of a validated variant, a false positive (FP) variant is defined as the detection of a nonvalidated variant, and a false negative (FN) is defined as the lack of detection of a validated variant by the pipeline. There are genuinely unique variants specific to a sample, which creates an underestimate in our scores. Since the problem applies to all pipelines, its effect can be discarded. 

**Figure 5:**
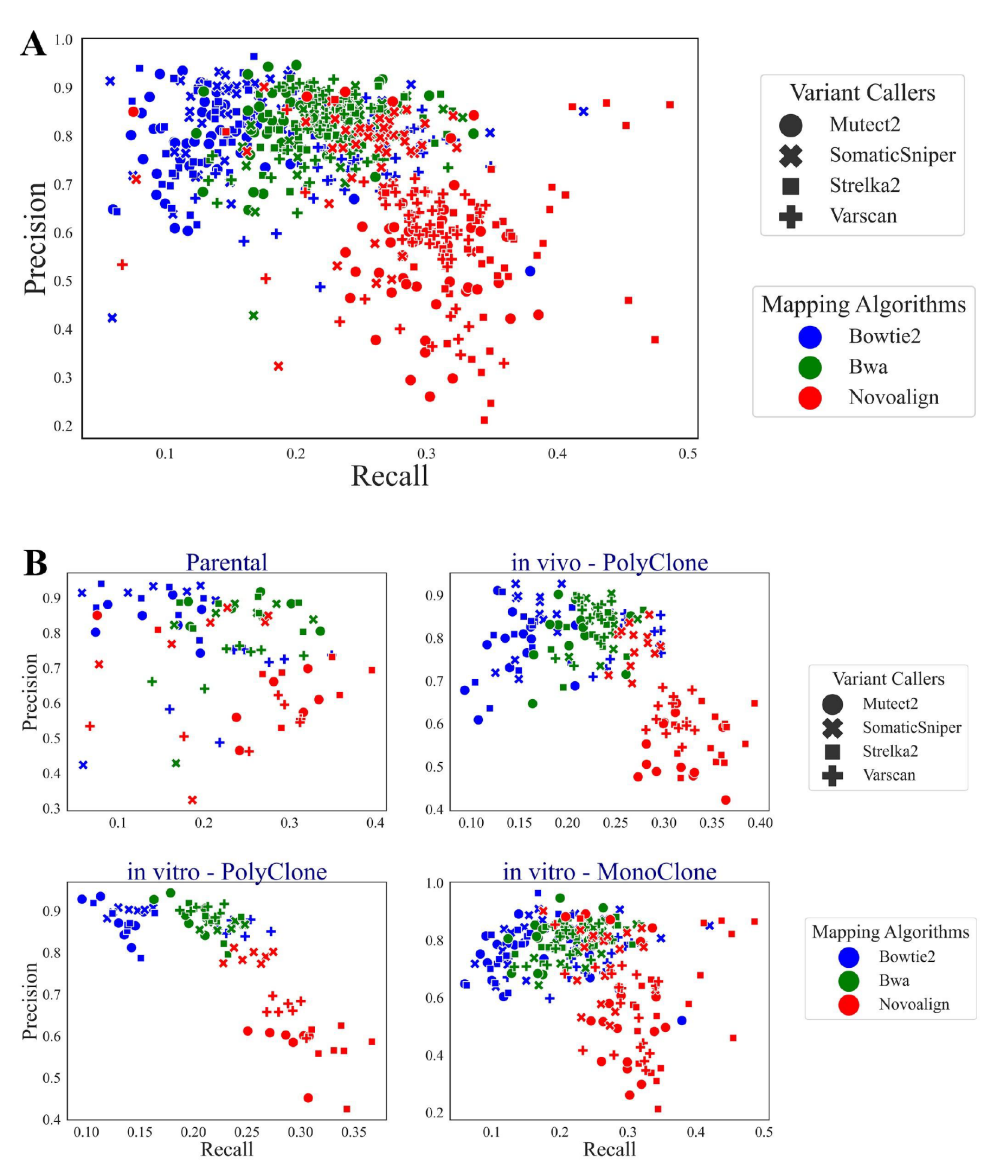
Scatter plots for precision-recall scores. Y-axis represents precision and X-axis shows recall. Colors indicate mapping algorithm choice while markers denote variant callers. (A) represents all the samples (46) with different pipelines (12) while (B) represents four subplots according to sample types.

We observed that pipelines that include Novoalign have high recall and less precision (Figures 4 and 5). In other words, both false positive and true positive counts are higher in these pipelines, which is concordant with the overall high number of detected variants in Novoalign pipelines. Quantitatively, pipelines that use Novoalign have recall scores around 0.35 while pipelines with Bowtie2 were around 0.15 and pipelines with BWA were around 0.25. This suggests that Novoalign pipelines capture significantly more variants out of all the validated variants. On the other hand, while BWA and Bowtie2 pipelines had a precision of ~0.90, meaning that 90% of their findings were validated variants while Novoalign performed worse with a precision of 0.65.

After evaluating the general patterns of algorithms, we focused on the individual pipeline performances with respect to recall and precision. Among the single pipelines, Novoalign-Strelka2 had the best recall score on most of the samples (44/46), while Bowtie2-SomaticSniper had the best precision score (20/46). Due to the low recall numbers for other algorithms, Novoalign-Strelka2 performed best in terms of F1 scores in 32 samples with ~0.39 on average (Table). 

**Table:** Top five pipelines according to F1 scores for three cases. After calculating every possible combination, a pipeline combination with the best F1 score is kept for each sample. Table includes the top five pipeline combination selections that have the most occurrences for 46 samples.

Pipeline #1	Pipeline #2	Pipeline #3	Parental	in vitro – MonoClone	in vitro – PolyClone	in vivo – PolyClone	Total
Novoalign_Strelka2	-	-	6	13	6	7	32
Bowtie2_Varscan	-	-	0	2	1	3	6
Novoalign_SomaticSniper	-	-	0	2	0	2	4
Novoalign_Varscan	-	-	0	1	0	1	2
Bwa_Mutect2	-	-	1	0	0	0	1
Bwa_SomaticSniper	-	-	0	1	0	0	1
Bowtie2_Varscan	Novoalign_SomaticSniper	-	0	5	3	6	14
Bowtie2_Varscan	Novoalign_Strelka2	-	0	4	3	3	10
Bwa_Mutect2	Novoalign_Strelka2	-	4	0	0	0	4
Novoalign_Varscan	Novoalign_Strelka2	-	0	1	1	2	4
Bowtie2_SomaticSniper	Novoalign_Strelka2	-	0	3	0	0	3
Bowtie2_Varscan	Novoalign_Strelka2	Novoalign_SomaticSniper	0	5	1	2	8
Bowtie2_Varscan	Bwa_Strelka2	Novoalign_SomaticSniper	0	1	2	3	6
Bowtie2_Varscan	Bwa_SomaticSniper	Novoalign_Strelka2	0	2	1	2	5
Bwa_Mutect2	Bwa_SomaticSniper	Novoalign_Strelka2	4	0	0	0	4
Bowtie2_SomaticSniper	Novoalign_Mutect2	Novoalign_Strelka2	0	4	0	0	4

### 3.1. Combination of pipelines

Each algorithm relies on different assumptions and has different priorities, resulting in different strengths and weaknesses in terms of variant detection. This makes the combination of different pipelines a potential solution for blending the strengths of different pipelines (Alioto et al., 2015; Hofmann et al., 2017). Since we obtained low recall scores and high precision scores, we hypothesized that we can improve the recall rates of individual pipelines by constructing the union of pipelines. Our aim was to find a point where recall and precision scores are balanced to achieve an optimal F1 score.

**Figure 6:**
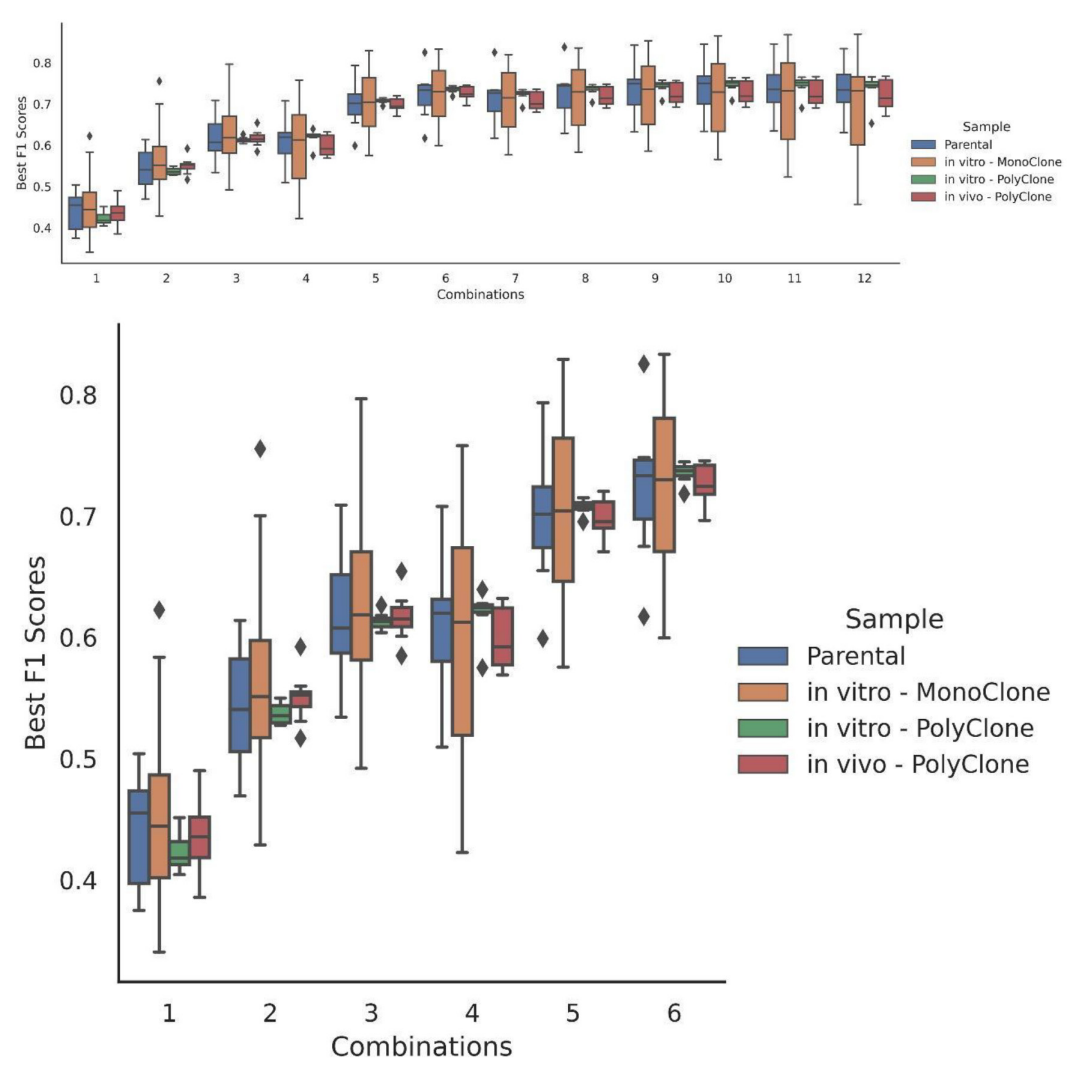
Distribution of the F1 scores depending on the number of pipelines used in combination. Colors indicate sample types and Y-axis represents the best F1 score while X-axis represents the combination selection. The first plot shows every combination ranging from using only one pipeline to using the union of all the 12 possible pipelines. The second plot is the clipped version until the combination of six.

We calculated the performance of every possible combination of different pipeline unions separately for each sample. Combinations that achieve the best F1 score for each sample were recorded. The most successful pipeline combinations are displayed on Table for (i) individual pipelines (ii) two pipeline combinations, and (iii) three pipeline combinations. Best pipeline combinations for every situation can be seen in Supplementary Table 2 and 3. As the number of pipelines in a combination increases up to six, F1 scores tend to get higher by a good margin (Figure 6). However, after six combinations, marginal loss on precision scores cannot be covered with recall gain. F1 scores are either insubstantially increased or slightly decreased as the combination number extends beyond six.

Our results show that F1 scores can be boosted up to around 0.7 with proper pipeline combinations. We found that Bowtie2_Varscan with Novoalign_Strelka2 / Novoalign_SomaticSniper can complement very well in monoclone and polyclone samples. Especially on polyclone samples, this combination outperforms every other combination in almost all samples. Although this combination produces the best results for most of the cases, primary tumor samples have a different pattern. BWA_Mutect2 and Novoalign_Strelka 2 combination performs better on primary samples. Overall, Bowtie2 and Novoalign pipelines complement very well on monoclones and polyclones while BWA and Novoalign pipeline unions are better choices on primary tumor samples.

### 3.2. Results on simulated variants

We simulated around 4800 mutations in three different purity environments to compare our results with the synthetic data where the real variants were known (materials and methods). In environments with low and high purity, we observed that variant caller choice has a strong effect on the number of variants found (Supplementary Figure 5). SomaticSniper finds far less mutations in low purity environment, while Mutect2 discovers fewer variants in high purity. Precision ratios were high in all pipelines with 99.99% accuracy in low-medium purity and 95+% in the high purity. On the other hand, recall ratios declined significantly as the purity of the environment decreased. Supplementary Figure 6 clearly demonstrates the huge variance in recall scores between the pipelines. Novoalign_SomaticSniper achieves 100% precision but with only 10% recall and Bwa_Strelka2 performs 99% precision with 75% recall scores. Strelka2 outperformed other variant callers on low purity environment with over 70% recall performance in all three aligner choices, where Varscan, Mutect2, and SomaticSniper only achieved 48%, 27%, and 10% on the average respectively. When variants of different pipelines were combined in the low purity environment, F1 scores increased considerably similar to the original samples (Supplementary Figure 7). These suggest that using a single pipeline results in highly variable outcomes depending on the variant caller chosen and combining multiple pipelines achieve better performance. Finally, we compared the similarity of the variants that were discovered in different pipelines. In this case, pipelines were clustered based on the variant caller similar to parental tumors and polyclonal samples (Supplementary Figures 8-10).

## 4. Discussion

Precision medicine relies on detailed profiling of patient samples. Recent developments in sequencing technologies allowed the measurement of DNA and RNA with an unprecedented resolution at dropping prices. This makes sequencing an ideal tool for studying genetic diseases such as cancer. Raw data obtained by massively parallel sequencing devices is large, error-prone, and highly redundant. It can be converted to useful information only with proper bioinformatics analysis, which makes best practices for analyzing sequencing data crucial for the effective utilization of sequencing technologies.

In this study, we evaluated the performance of the most popular cancer sequencing pipelines. We used high-resolution heterogeneous and homogeneous samples that belong to a single tumor to measure the performances of sequencing algorithms for different heterogeneity levels in a realistic scenario (as an alternative, we could have used single-cell sequencing, but resolution drops significantly due to amplification). Cancer sequencing analysis consists of two major steps, namely mapping and variant discovery. In our work, we used the most popular algorithms that we could obtain for both steps. After selecting three mapping and four variant discovery algorithms, we constructed and evaluated twelve pipelines as the combinations of these algorithms to assess the coherence between different mapping and variant discovery algorithms.

Sequencing algorithms can usually be executed with different parameters to give users the opportunity to adapt the algorithms to different scenarios. We used the default or recommended parameters for each algorithm to assess their general performance. This created a slight problem for our comparisons since certain pipelines had found more variants than others. Especially pipelines that include Novoalign had more variants, while pipelines that used Bowtie2 had fewer variants. We could change some parameters to make the count of identified variants more similar, but we preferred to keep the recommended parameters to keep our comparison more practical since most of the users will prefer default settings.

An important and distressing result was the limited overlap between the variants that different pipelines discover. Even with most homogeneous samples that were cultured from a single cell, we observed a limited overlap (as low as 50%), which matches up with the results of similar study reporting an inter-caller agreement rate around 50% on exome samples (Krøigård et al., 2016). We tried to use commonly identified variants in different samples as a metric of correctness since a false discovery of the identical variant in different samples is unlikely. We defined these commonly detected variants as “validated variants” and computed recall and precision rates for each pipeline based on these variants. We observed a clear precision and recall trade-off among pipelines. Pipelines that report more variants demonstrated higher recall but lower precision rates. To quantify the marginal gain of reporting extra variants we calculated the F1 scores for each pipeline based on recall and precision rates, which comes out to be lower than 0.5 for most of the pipelines. Previous studies have shown that combining outputs of several variant callers increases performance on detecting variants and amplifies the F1 score (Rashid et al., 2013; Kim et al., 2014). Similar results also appeared in our study in which higher F1 scores could be obtained by combining (union) identified variants of different pipelines. We did extensive experiments to determine the pipeline combinations that produced the highest F1 score. Our results indicate that combinations that include five or six pipelines with complementary algorithms (such as Bowtie2-Varscan and Novoalign-Strelka2) perform best for identifying variants.

Many different benchmarking studies have been conducted to assess the accuracy of somatic variant callers or aligners. Nevertheless, the heterogeneity of cancer samples can affect the results considerably. Therefore, in this study, we aimed to perform a benchmarking study of both different variant callers and aligners in different heterogeneity levels on real and simulated datasets. After illustrating the benchmark results, we proposed using a combination of different tools to utilize the somatic variant calling pipelines’ performances.

For the evaluation of mutations, we declare the mutations that were discovered by only a single pipeline as false positives. We are aware that some real variants might be captured only by a single pipeline and thus may not be real false positives. However, this was a very unlikely event considering very high precision rates and low number of false positive mutations, which was concordant with detailed sampling of a single tumor (600 different runs based on the same tumor (12 pipelines × 50 samples)). Therefore, increasing the recall score by combining variants, which are found by different pipelines, would not be affected by any misclassified false positives as our method proposes. Furthermore, our analysis of the simulated dataset suggests that recall scores are poor on low heterogeneity samples similar to the real dataset.

This work presents a framework for extensive analysis of cancer sequencing pipelines. We plan to use this framework on different data sets in future studies to have a better understanding of pipeline performance in different practical scenarios. The framework software is available on GitHub2. We invite all interested parties to extend our work.

## Supplementary Data

Supplementary data can be accessed at the following link: https://dx.doi.org/biy-2008-8-sup
